# Oocyte specific lncRNA variant *Rose* influences oocyte and embryo development

**DOI:** 10.1016/j.ncrna.2021.06.001

**Published:** 2021-06-26

**Authors:** Rajan Iyyappan, Daria Aleshkina, Linkai Zhu, Zongliang Jiang, Veronika Kinterova, Andrej Susor

**Affiliations:** a Laboratory of Biochemistry and Molecular Biology of Germ Cells, Institute of Animal Physiology and Genetics of the Czech Academy of Sciences, Rumburska 89, 277 21, Libechov, Czech Republic; b School of Animal Sciences, AgCenter, Louisiana State University, Baton Rouge, LA, 70820, United States; c Laboratory of Developmental Biology, Institute of Animal Physiology and Genetics of the Czech Academy of Sciences, Rumburska 89, 277 21, Libechov, Czech Republic

**Keywords:** LncRNA, Oocyte, Early embryo, Polysome, Meiosis

## Abstract

Fully grown mammalian oocytes store a large amount of RNA synthesized during the transcriptionally active growth stage. A large part of the stored RNA belongs to the long non-coding class which contain either transcriptional noise or important contributors to cellular physiology. Despite the expanding number of studies related to lncRNAs, their influence on oocyte physiology remains enigmatic. We found an oocyte specific antisense, long non-coding RNA, “*Rose”* (*lncRNA in Oocyte Specifically Expressed*) expressed in two variants containing two and three non-coding exons, respectively. *Rose* is localized in the nucleus of transcriptionally active oocyte and in embryo with polysomal occupancy in the cytoplasm. Experimental overexpression of *Rose* in fully grown oocyte did not show any differences in meiotic maturation. However, knocking down *Rose* resulted in abnormalities in oocyte cytokinesis and impaired preimplantation embryo development. In conclusion, we have identified an oocyte-specific maternal lncRNA that is essential for successful mammalian oocyte and embryo development.

## 1 Introduction

Long non-coding RNAs (lncRNAs) are stretches of RNA of at least 200 nucleotides which are not translated into protein. The vast majority of human and mouse transcriptome belongs to this noncoding class. Breschi et al., 2017 annotated 15,767 and 9989 lncRNAs in the human and the mouse [1]. Whilst the major part of all transcribed RNA belongs to ncRNA, they have not been well characterized so far. ncRNAs present in various tissues and cells are mostly alternatively spliced or processed into smaller RNA [2]. Recent evidence shows that lncRNAs are engaged in all aspects of cellular activity with lncRNAs predominantly playing specific roles inside the nucleus and regulating transcriptional and posttranscriptional processes [3,4], as well as epigenetics [2]. Moreover, accumulating evidence shows that lncRNAs form complexes with diverse structural and regulatory functions in the cytoplasm along with RNA binding proteins and mRNAs [5]. LncRNAs display different subcellular localization and possess distinct regulatory impacts at their particular site of action [6,7].

Although there have been studies into the functions of lncRNAs in mammalian cells, their roles in germ cells are largely unknown. Recently there were just a few studies stating the importance of lncRNA in germ cells [8,9] and its evolutionary significance [10]. Fully grown mammalian oocytes store a large amount of RNA synthesized during the transcriptionally active growth stage, most of which belongsto a non-coding class, contributing to cell physiology, and yet, also merely transcriptional noise.

In this study, we characterised mouse *lncRNA in Oocyte Specifically* *Expressed* (“*Rose”*) in the mouse oocyte and early embryo. We investigated the expression and localization of *Rose* at the various stages of oocyte and early embryo development. Moreover, we elucidated the function of *Rose* by gain- and loss-of-function approaches in order to study its contribution to cell physiology.

## 2 Materials and methods

### 2.1 Oocyte isolation and cultivation

The females of 6-week-old ICR mice were stimulated with 5 IU pregnant mare serum gonadotropin (PMSG; Folligon; Merck Animal Health) per mouse. After 46 h, the oocytes were isolated from the ovaries. Fully grown germinal vesicle (GV) oocytes were isolated into transfer medium (TM) supplemented with 100 μM 3-isobutyl-1-methylxanthine (IBMX; Sigma Aldrich) for the prevention of spontaneous meiotic resumption. Selected oocytes were denuded and cultivated in M16 medium (Millipore) without IBMX at 37 °C, 5% CO_2_ for 0 h (GV) or 16 h during second metaphase arrest (MII). For embryo collection, the stimulated mice were again injected with 5 IU hCG before being mated overnight with males of the same strain. After 16 h, zygotes were recovered from the excised oviducts and cultured in EmbryoMax Advanced KSOM Embryo Medium (Sigma-Aldrich).

All animal experiments were performed in accordance to guidelines and protocols approved by the Laboratory of Biochemistry and Molecular Biology of Germ Cells at the Institute of Animal Physiology and Genetics in Czech Republic. All animal work was conducted according to Act No. 246/1992 on the protection of animals against cruelty, issued by experimental project #215/2011, certificate #CZ02389, issued by the Ministry of Agriculture.

### 2.2 PCR and RT-PCR

RNA was extracted using TRI reagent (Sigma). The equal amount of RNA was used for cDNA synthesis using both hexamers and oligo-d(T) primers (qPCR BIO cDNA Synthesis Kit, PCR Biosystems). For PCR (PPP master mix, TOP-Bio) the following program was used: 94 °C 5 min; 94 °C 15 s; 58–60 °C 15 s; 72 °C and then the products were separated on 0.8% agarose gel with GelRed (41003, Biotinum) staining. RT-PCR (Luna Universal qPCR Master Mix, New England BioLabs) was carried out using QuantStudio3. qPCR data were normalized to GAPDH expression by the ΔΔCt approach. Primers are listed in Supplementary Table 1. RNA extraction, PCR and RT-PCR were all performed according to the manufacturer's instructions.

### 2.3 RNA FISH

RNA FISH was performed following Tetkova et al. [11]. Briefly: oocytes were fixed (15 min in 4% PFA) and pre-treated with protease III (diluted 1:15 in nuclease-free water; Cat. No. 322381, ACD) for 10 min. Each sample was then incubated with corresponding RNAScope probes (Supplementary Table 1) at 2 h in 40 °C to detect *Rose*. RNA FISH protocol for amplification was followed using RNAScope Multiplex Fluorescent Detection Reagents v2 kit (Cat. No. 323110, ACD), with extended washing. After amplification, HRP-C1/C2/C3 was used on the corresponding channels of specific probe, for 15 min, 40 °C. Oocytes were washed again 2 × 5 min in 1x wash buffer. TSA Cy5 dye (PerkinElmer) diluted to 1:1500 in TSA buffer (ACD) was used for fluorescent labelling of the amplified signal. After washing and application of HRP blocker (30 min in 40 °C), samples were washed a final time 2 × 5 min in 1x wash buffer and mounted in Prolong Gold Antifade with DAPI (Life Technologies) on epoxy coated slides (Thermo Scientific). Images were obtained using a confocal microscope (Leica SP5). Image quantification of single equatorial Z was performed by ImageJ software (http://rsbweb. nih.gov/ij/). Images were converted to the binary type and threshold range was set to distinguish fluorescent RNA signals from the background. Quantification was performed via standard ‘Analyze particles’ tool. Bacterial *DapB* RNA (*Bacillus subtilis*, str. SMY; EF191515.1) was used as a negative control.

### 2.4 Polysome fractionation

Polysome fractionation followed by RNA isolation was carried out according to the Scarce Sample Polysome profiling (SSP-profiling) method by Masek et al. [12]. Briefly, at the time of oocyte collection, 200 oocyte/embryos were treated with 100 μg/mL cycloheximide for 10 min and collected in 350 μL lysis buffer (10 mM Hepes, pH 7.5; 62.5 mM KCl, 5 mM MgCl2, 2 mM DTT, 1% TritonX-100) containing 100 μg/mL CHX and 20 U/ml Ribolock (Thermo Fisher Scientific). After disruption of the zona pellucida with 250 μL of zirconia-silica beads (BioSpec), lysates were centrifuged at 8000 g for 5 min at 4 °C. Supernatants were loaded onto 10–50% sucrose gradients. Centrifugation was performed at 45,000 RPM (246,078×*g*) for 65 min at 4 °C (Optima L-90 ultracentrifuge, Beckman Coulter). Ten equal fractions were collected from each polysome profile and subjected to RNA isolation. These RNA and its profile were validated using the primer for 18s and 28s rRNA by qPCR [12]. Then, non-polysomal (NP; fractions 1–5) and polysomal fractions (P; fractions 6–10) were pooled and subjected to qRT-PCR (QuantStudio 3 cycler, Applied Biosystems) using *Rose* NCE1 specific primers.

### 2.5 Immunocytochemistry

Oocytes were fixed (15 min in 4% PFA; Sigma Aldrich), permeabilized (10 min in 0.1% Triton X-100) and washed in PBS supplemented with polyvinyl alcohol (PVA, Sigma Aldrich) and incubated with anti-acetylated α-tubulin (T7451, Sigma Aldrich) diluted in PVA/PBS, overnight at 4 °C. Oocytes were then washed 2 × 15 min in PVA/PBS and primary antibodies were detected using relevant Alexa Fluor 488/594/647 conjugates (Invitrogen) diluted to 1:250 for 1 h at room temperature. Washed oocytes (2 × 15 min in PVA/PBS) were mounted onto slides using Vectashield with DAPI. An inverted confocal microscope (Leica SP5) was used for sample visualization. Morphology of the spindles (anti-acetylated α-tubulin) and chromosomes (DAPI) were defined by spindle morphology and chromosomal alignment. Spindles were analysed as maximum intensity projection Z-stack images using LAS X (Leica) software. Experiments were repeated 3x with 20–30 oocytes per group/experiment.

### 2.6 *Invitro* transcription, microinjection and live-cell imaging

H2b:*gfp* RNA from plasmid (provided by Dr Martin Anger, Laboratory of Cell Division Control, IAPG CAS) and *Rose* cRNA for overexpression was prepared using T7 mMessage, Ambion kit. The dsRNA against *Rose* was prepared using a MEGAscript RNAi Kit. These dsRNA were digested by ShortCut^R^ RNase III (New England Biolabs) for making small and efficient dsRNA [13]. As a negative control, we used MISSION® esiRNA (control) targeting Renilla luciferase (RLUC, Sigma Aldrich).

Isolated fully grown oocytes/Zygotes were microinjected in TM with/without IBMX using a Leica DMI 6000B inverted microscope, TransferMan NK2 (Eppendorf) and FemtoJet (Eppendorf). Solution used for oocyte/embryo injection contained: 20 ng/μL of *in vitro* transcribed *H2b:gfp* RNA in combination with 100 ng/μL (overexpression) or 1000 ng/μL esiRenila (dsRenilla) or dsRose. 24 h after microinjection, oocytes were washed from IBMX and cultivated to MII stage. In case of zygotes, after 4 h of microinjection, the embryos were transferred into KSOM (Sigma-Aldrich, Merck) media for further development. Microinjected oocytes were placed into a 4-well culture chamber (Sarstedt) in 10 μL of equilibrated M16 media (37.5 °C, 5% CO_2_) covered with mineral oil (M8410; Sigma Aldrich). The cells were imaged using a Leica DMI 6000B inverted microscope equipped with a controlled chamber system (Temp controller 2000–2 Pecon, and a CO_2_ controller, Pecon). Time lapse recordings (LAS X, Leica microsystems) of meiotic maturation of microinjected oocytes were used for phenotype evaluation (nuclear envelope breakdown, polar body extrusion).

### 2.7 *In silico* prediction

RNA-RNA interactions were predicted by using the IntaRNA tool with default settings (http://rna.informatik.uni-freiburg.de/IntaRNA/Input.jsp) [14]. Results are presented in Supplementary Table 3. Noncoding potential analysis was predicted using the Coding Potential Assessment Tool (CPAT) (http://lilab.research.bcm.edu/cpat/) [15].

### 2.8 Statistical analysis

Experiments were repeated at least 3 times unless stated. Mean and SD values were calculated using MS Excel, statistical significance of the differences between the groups was tested using Student's t-test and we applied one way ANOVA for comparisons of more than two groups then Tukey's multiple comparisons test as a post-hoc test (PrismaGraph5). p < 0.05 was considered as statistically significant.

## 3 Results

### 3.1 *Rose* lncRNA variant expressed only in the mouse oocyte and early embryo

**Fig. 1:**
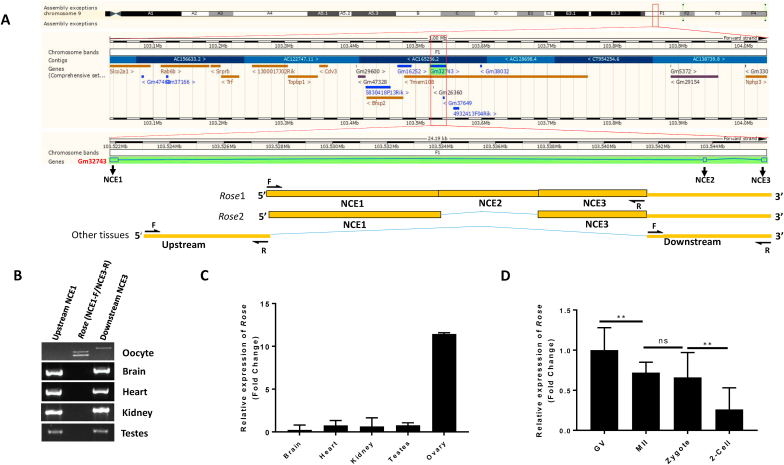
*Rose* lncRNA variant expressed only in the mouse oocyte and early embryo. (A) Scheme of genome organisation of *Gm32743* from Ensembl browser. Also see Supplementary Fig. 1. **(B)** PCR detection of *Rose* lncRNA in oocyte and mouse tissues. Also see Supplementary Fig. 2. **(C)** qRT-PCR detection of *Rose* expression in various mouse tissues. Also see Supplementary Fig. 4A. **(D)** Expression of *Rose* lncRNA in GV, MII, zygote and 2-cell stage embryo. Mean ± SD; One-way ANOVA: F (2, 3) = 66.07, p < 0.01. Tukey's multiple comparisons test: **p < 0.01, ns - non-significant; n = 2. Also see Supplementary Fig. 4A.

**Fig. 2:**
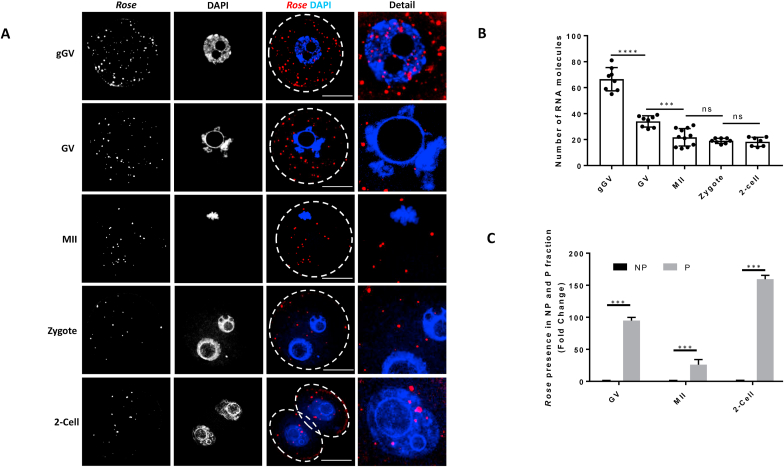
Cytoplasmic localized *Rose* is present in the polysomal fraction in oocyte and early embryo. (A) Localization of *Rose* lncRNA detected by RNA FISH in growing and fully grown oocytes and early embryo. Combination of single equatorial optical section for *Rose lncRNA* and maximum intensity projections for DAPI. Representative images from three biological experiments. Scale bar = 25 μm *Dab8* RNA was used as a negative control Supplementary Fig. A and B. (**B)** Quantification of *Rose* lncRNA molecules in one Z section from RNA FISH. Mean ± SD; One-way ANOVA: F (4, 37) = 99.15, p < 0.0001. Tukey's multiple comparisons test: ****p < 0.0001, **p < 0.01, ns - non-significant; from three biological replicates, n ≥ 8. **(C)** qRT-PCR detection of *Rose* in the non-polyribosomal (NP) and polyribosomal (P) fractions in the oocyte and early embryo. Mean ± SD; Student's t-test: ***p < 0.001; n = 3.

The gene coding for *Gm32743* is located on chromosome 9 and is transcribed as linear, antisense RNA 1611 nucleotides (nt) in length (Fig. 1A). According to the mouse ENCODE database, *Gm32743* lncRNA is present in almost all mouse tissues and is highly expressed in the heart and brain (Supplementary Fig. 1). *Gm32743* contains three non-coding exons: NCE1 (312 nt), NCE2 (112 nt), and NCE3 (170 nt) (Fig. 1A and Supplementary Figs. 2A–C). We found that only the oocyte and embryo expresses two variants of *Rose* lncRNA (Fig. 1A and B) which contains exons NCE1-3 (variant 1) and exons NCE1&3 (variant 2) (Fig. 1A and Supplementary Figs. 2A and B), respectively. Interestingly, our semi quantitative and qPCR data shows that neither variants of *Rose* are found in other mouse tissues (Fig. 1B and C). However, upstream NCEs exist only in other tissues whereas downstream NCEs have been found in all analysed tissues, including oocytes (Fig. 1B). Alignment of *Gm32743* showed no significant similarity with other organism. Next, we analysed the expression of both *Rose* lncRNA variants using primers specific to NCE1 in the fully grown GV, matured MII oocyte and 1- & 2-cell embryo. We found that *Rose* has the highest expression in the GV oocyte with a significant decrease in the 2-cell embryo (Figs. 1D and 2A and B). In order to exclude possibility of genomic DNA contamination in the samples, as a control, *Dazl* exon 3 and 4 specific primers were used to amplify the expected PCR product (Supplementary Fig. 3).

In conclusion, we found that *Gm32743* is spliced and its variant exclusively expressed in the mouse oocyte and early embryo generating *Rose* lncRNA.

### 3.2 Cytoplasmic localised *Rose* is present in the polysomal fraction in oocyte and early embryo

As it might predict the RNA's role in the cell [16], we examined the localization of *Rose* in the oocyte and early embryo. Using RNA FISH approach, we found that the transcriptionally active growing oocyte (growing GV; gGV) and 2-cell embryo have *Rose* lncRNA distributed in both the nucleus and the cytoplasm (Fig. 2A and B). Contrastingly, *Rose* was not present in the nucleus of the transcriptionally inactive fully grown GV oocyte and Zygote (Fig. 2A and Supplementary Fig. 4A). Similar to the qRT PCR analysis, RNA FISH showed a significant decrease of *Rose* in the matured MII oocyte, zygote and 2-cell embryo (Figs. 1D and 2A and B). As a negative control for RNA FISH we used a probe specific for bacterial RNA *Dab8* and it was not detected in oocytes and early embryos (Supplementary Fig. 4B). Previously we detected ncRNA in the cytoplasm and polysomal fractions [17] so we asked if *Rose* is present in non-polysomal (NP) and polysomal (P) fractions from fully grown GV, MII oocytes and 2-cell embryos. Interestingly, we found that *Rose* was enriched in the polysomal fraction which was confirmed by qRT PCR (Fig. 2C). *Rose* is annotated as lncRNA, however we detected its polysomal occupancy. Thus we asked if *Rose* has coding potential. Analysis by the Coding Potential Assessment Tool (CPAT) produced a negative hexamer score (−0.187956336; Supplementary Table 2), confirming the non-coding nature of *Rose*. As a positive control, *Xist lncRNA* and *Cyclin B1* mRNA were analysed with known noncoding *Xist* [18] and protein coding *Ccnb1* [10] (Supplementary Table 2). Moreover *in silico* RNA-RNA interaction prediction analysis shows the positive interaction of *Rose* with noncoding and protein coding RNAs (Supplementary Table 3).

Here, we found that *Rose* is present in the nucleus of transcriptionally active growing oocytes and early embryos. Furthermore, despite *Rose* having no translational potential we detected it in the polysomal fraction.

### 3.3 Downregulation of *Rose* leads to aberrant meiotic progression and early embryo development

**Fig. 3:**
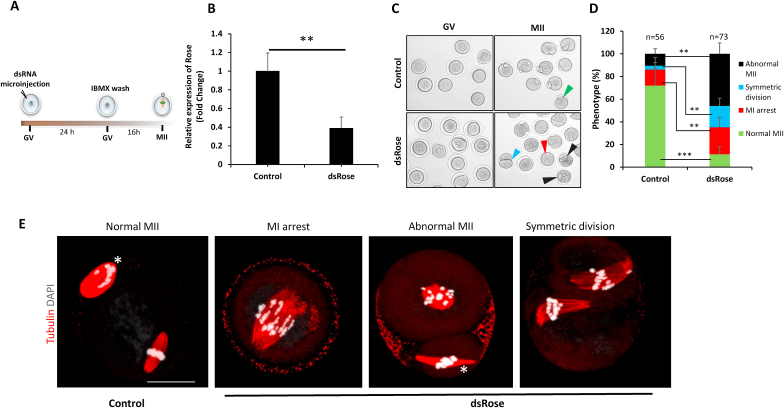
Downregulation of *Rose* leads to aberrant meiotic progression. (A) Scheme of experimental approach for *Rose* downregulation in the oocyte. **(B)** qRT-PCR detection of knock down of *Rose* using dsRNA. Mean ± SD; Student's t-test: **p < 0.01; n = 3. **(C)** Phenotype analysis of progression of GV oocytes to MII stage after downregulation of *Rose* lncRNA. Arrowheads (except green) depict aberrant meiotic progression. **(D)** Quantification of oocyte progression from GV to MII stage after downregulation of *Rose* lncRNA. Mean ± SD; Student's t-test: ****p < 0.0001, **p < 0.01; from three biological replicates with presented *n*. *dsRenila* was used as a control**. (E)** Representative oocyte morphologies of oocytes microinjected with *dsRenilla* (control) and *dsRose*. Tubulin red and chromosomes labeled by DAPI (Gray); scale bar 10 μm; asterisk depicts polar body. (For interpretation of the references to colour in this figure legend, the reader is referred to the Web version of this article.)

**Fig. 4:**
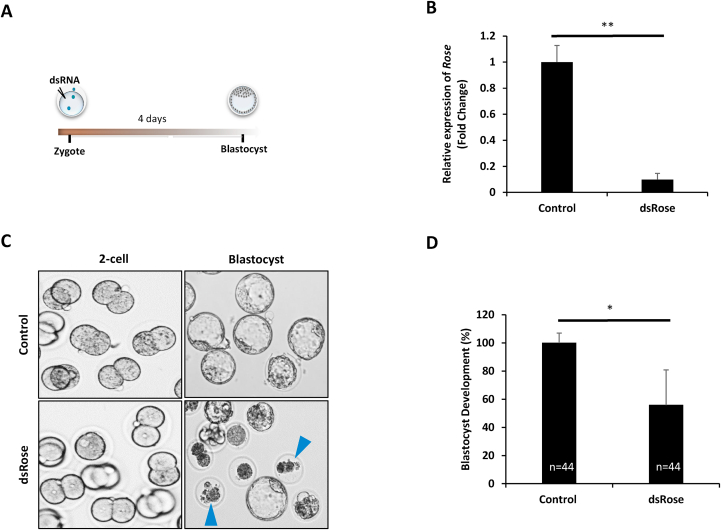
Downregulation of *Rose* affects early embryo development. (A) Scheme of experimental approach for *Rose* downregulation in the embryo. **(B)** Knock down of *Rose* using dsRNA. qRT-PCR result of 2-cell embryos of control and dsRNA injected zygotes. Mean ± SD; Student's t-test: **p < 0.01; n = 3. **(C)** Phenotype analysis of progression of blastocyst stage after downregulation of *Rose* lncRNA. Arrowheads depict fragmented embryos after 2-cell stage. **(D)** Quantification of blastocyst development after downregulation of *Rose* lncRNA. Mean ± SD; Student's t-test: *p < 0.05; from three biological replicates with presented *n*.

To further investigate the role of *Rose* in the oocyte and early embryo physiology, we performed overexpression of *Rose* by microinjection into the GV oocyte (Supplementary Fig. 5A) leading to its significant increase (Supplementary Fig. 5B). Following time lapse observation, no abnormalities were found in the oocyte meiotic progression (Supplementary Figs. 5C and D). Next, we performed knockdown (KD) of *Rose* in the GV oocyte (Fig. 3A and B). Here meiotic progression was quantified based on polar body extrusion. Time lapse imaging shows that 88.6% of the oocytes exhibited significant abnormal meiotic progression in response to *Rose* downregulation, which is 60.7% higher than the *dsRenilla* injected control (27.95%) (Fig. 3C–E). Oocytes in both groups underwent nuclear envelope breakdown normally, however in presence of *dsRose* majority of oocytes failed to extrude a polar body which led to abnormal MI (red arrow head), abnormal polar body extrusion and symmetrical division (Fig. 3D and E). Moreover 64.7% of oocytes with extruded polar body showed irregularities in spindle and chromosome organisation (Fig. 3E). Finally, we investigated whether downregulation of *Rose* influences embryo development by *Rose* KD in the zygote (Fig. 4A and B). We found no significant differences in the progression to the 2-cell stage in either group (Supplementary Fig. 6) however the blastocyst rate was significantly lower (44.21%) in the *Rose* downregulated group compared to control (Fig. 4C and D). In addition to this, we observed that embryos were arrested at the 2–8 cell stage (Fig. 4C).

In conclusion, we found that maternal *Rose* lncRNA has a significant role in the meiotic progression of the oocyte as well as in embryo development.

## 4 Discussion

Emerging RNA-seq technology and transcriptome analyses have uncovered a growing number of lncRNAs and their regulation over protein-coding in various cells and animal species. However, functional analysis of lncRNAs is still challenging, and so far the molecular role has only been explored for a small subset of lncRNAs. Majority of lncRNAs are just transcriptional noise only some contributing to cellular physiology. Annotation of mouse maternal lncRNAs has revealed a number of lncRNAs, but their roles still remain enigmatic.

The oocyte signature includes functionally recognized oocyte-specific mRNAs such as *Oog1* [19], *Dazl* [20], *ZP1-3* [21], *Figla* [22], and *Gdf-9* [23]. However, oocyte-specific lncRNAs are not well known and have no recognized role in the oocyte. We discovered Rose *(lncRNA in Oocyte Specifically Expressed)*, a maternal lncRNA uniquely transcribed and processed in the mouse female germ cell. LncRNAs are poorly conserved compared to protein coding RNAs and most are expressed specifically in particular cells/species [24]. Similarly, *Rose* lncRNA did not share any detectable similarity with lncRNAs in other species, suggesting that *Rose* appeared after mouse split from its ancestor.

Interestingly, in transcriptionally silent fully grown oocyte, *Rose* is localized only in the cytoplasm, however, in transcriptionally active growing oocyte and 2-cell embryo, *Rose* exhibits in both nucleus and cytoplasm. For many lncRNAs subcellular location is directly linked to their function [7,25] and the nucleus and cytoplasm are well defined barriers for gene expression such redistribution of *Rose* is suggesting cell stage specific regulatory mechanisms. Nuclear localization of *Rose* in relation with transcriptional activity might contribute to transcription associated processes, epigenetic regulation and/or RNA transport. Detected *Rose* molecules in the nucleus do not constitute transcriptional hotspots which represent one or two large spots in the chromatin [26].

Moreover, previously was shown that lncRNA can regulate target genes on both epigenetic and translational levels [27,28]. This regulations often involve significant degree of complementarity between lncRNA and mRNAs which can link role of *Rose* with metabolism of target mRNAs leading to observed polysomal association and impact on translational regulation.

The specific spatio-temporal expression and localization can be linked to the establishment of both transcriptional and post transcriptional processes which might connect *Rose* with polysomal occupation or ribosomal protein maturation [29]. Similarly, *BC1* ncRNA was detected in the polysomal fraction from GV oocytes. *BC1* ncRNA is an example where the Fragile X Mental Retardation Protein (FMRP) is a co-player of ncRNA to promote translational repression in the cell [17]. Moreover, lncRNAs can physically interact with ribosomes or via recruitment of specific transcripts to the ribosome machinery [30]. Such a versatile nature of lncRNAs, as evidenced in recent studies, is in close corroboration with *Rose.* We presume that the localization in the transcriptionally active nucleus combined with RNA-RNA interaction and polysomal presence indicate multi-mode action of *Rose* in RNA fate in the development of oocyte and early embryo.

Aberrant meiotic spindle in *Rose* downregulation, the one might predict the aberrancies in embryo cleavage. However, there was no arrest or malfunction in the cleavage from one-cell embryo to a two-cell embryo. Conversely, absence of *Rose* leads to detrimental effect on embryonic development post 2-cell stage. In addition, we discovered that the *Rose* is localized in the nucleus of the two-cell stage indicating role in the nucleoplasm of transcriptionally active cell. In conclusion *Rose* has possible different functions in oocyte maturation and early embryo development. Based on the observed phenotypes and oocyte-zygote expression, we hypothesize that *Rose* has a specific role in the female germ cell and consequently in the early embryo development. Diverse molecular and biological roles have been assigned to lncRNAs, although most of them probably did not acquire a detectable biological role under laboratory conditions e.g. *Neat2*, *Sirena1* [10,31]. Moreover, we found that maternal effect *Rose* lncRNA has an essential role in the achievement of meiotic and zygotic developmental competence.

Overall, *Rose* lncRNA has an important regulatory role in oocyte cytokinesis and the post maternal-to-zygotic transition in early embryo development. However, further study is required to explore the specific role of the *Rose* lncRNA in the development of the mouse oocyte and embryo.

## Ethical approval

All animal work was conducted according to Act No 246/1992 for the protection of animalsagainst cruelty; from 25.09.2014 number CZ02389, issued by the Ministry of Agriculture.

## Funding

This research was funded by GACR18-19395S and Institutional Research Concept RVO67985904. Z.J. was supported by the funds from the NIH (R01HD102533) and USDA-NIFA (2019-67016-29863). The funders had no role in study design, data collection and analysis, decision to publish, or preparation of the manuscript.

## CRediT author statement

R.I. designed the experiments, carried out the data analysis and planed the project. carried out most of the experiments. wrote the manuscript. A.S. wrote the manuscript. designed the experiments, carried out the data analysis and planed the project; D.A. performed and analysed RNA FISH; L.Z. performed polysomal data analysis. Z.J. performed polysomal data analysis; V.K. designed and prepared dsRNA. All authors edited the manuscript.

## Declaration of competing interest

The authors have declared that no competing interests exist.
