# Supplementary material for: Oocyte specific lncRNA variant Rose influences oocyte and embryo development
Source: Noncoding RNA Res. 2021 Jun 26;6(2):107–13. doi: 10.1016/j.ncrna.2021.06.001 (PMC8258604; doi:10.1016/j.ncrna.2021.06.001)
Supplement: Multimedia component 1 [file mmc1.pdf]

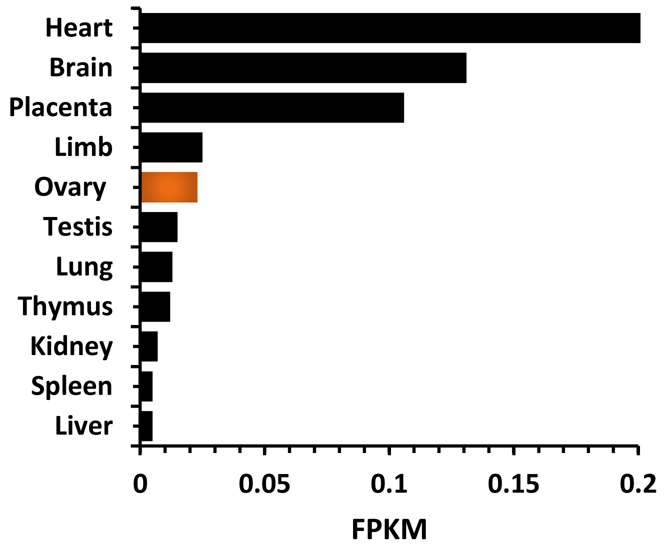

Supplementary Fig. 1: *Gm32743* lncRNA expression in mouse tissues. Expression estimated as FPKM from Mouse ENCODE transcriptome data.

A

No.	Non coding Exon	Start	End	Length	Sequence
NCE1	ENSMUSE00001394345	10,35,21,787	10,35,22,098	312	TCCAGGTTCTTCTTACAATGTGACGCAGATCTATGGACAGAGTCTGTGTTCCTGACCCTT GTATCTGTGACTACGGCAGGAGTGACCCTAAGTTCCAAGGCTAGGTCAGGAGTGAAGATG TGTCATAAGTCTGGGTGGAGCTATATCATACAGAGGTGGCTGGACAGCCCGGGGTGCACT TTTCCCAGAAGTAACTGCCAGACGTTCAAGTGTGAAAACCTTCAGCTGACTTCAGCTGGC ACGAACAGTTCTCCTTCACCCGAGCAAATCCGACGGACCTCACTACAGACTGGGCTGTGG CGTATTTTTCAT
NCE2	ENSMUSE00001397623	10,35,43,642	10,35,43,753	112	GTACCAGAGTGCCACCATTGATCACAGCTCTAAGAGGGTGTTTGTGAAGTCAGGAAGTAG TATTCCACGCCTTTAATTCCAGCACTCAGGAGGCAAAGACAGGAGGATTTCT
NCE3	ENSMUSE00001398719	10,35,45,808	10,35,45,977	170	AGACAGCCCCCTCCCCCTTTGGCTGCAGCTTCAGCAGTGCTTGAGTTTGGTGCAACCAAC CCATGGATGAGGCTTCTTGGGGGAAGCTGTGACCCTGGAAGAGGCAGGGAGGGAGAAG GCTGCGGTGACTCATGCTTCCAACTGGACTGGCTCATCAAGTCTGTTTTTGG

B

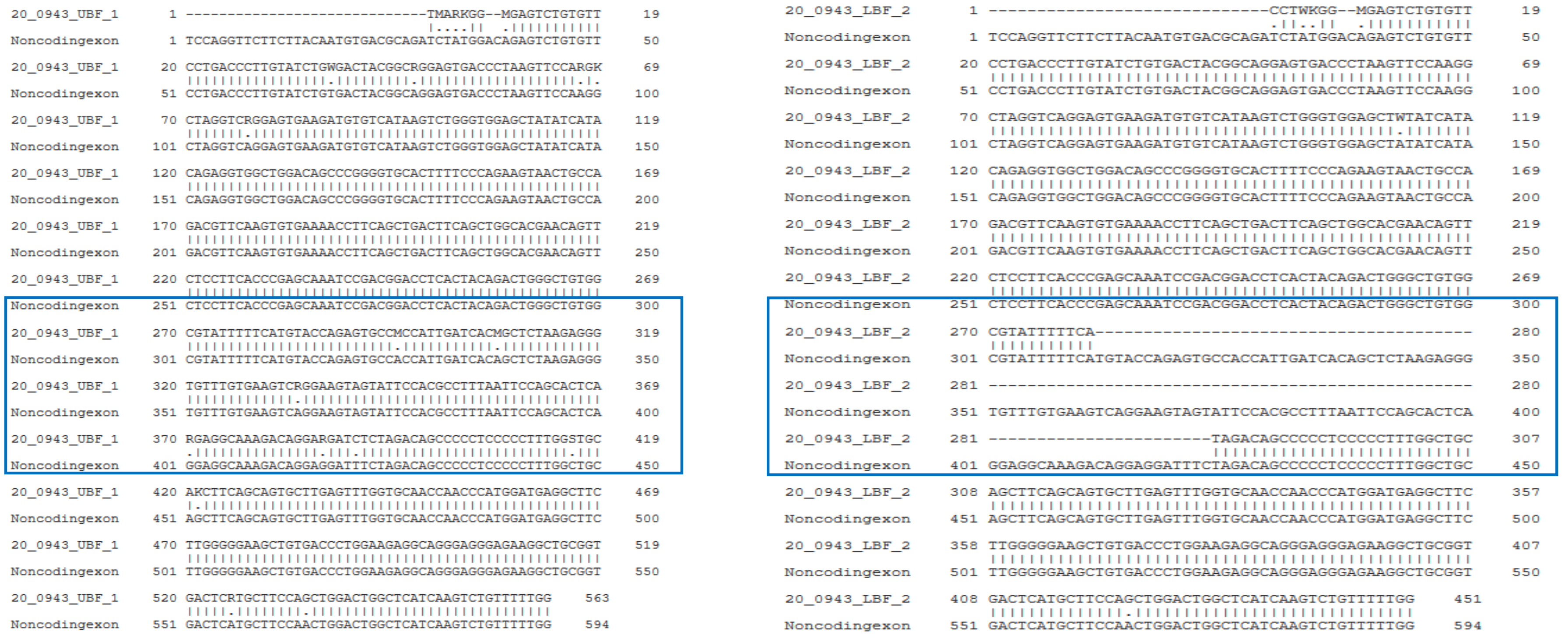

Rose NCE 1+2+3 – Upper Band

Rose NCE 1+3 – Lower band

C

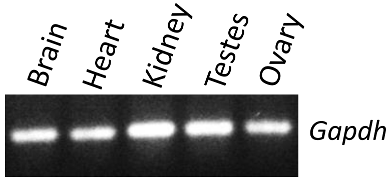

Supplementary Fig. 2: Sequence details of non-coding-exons in *Rose*. (A) Non-coding exon region of *Rose* (ENSMUST00000214196.1) from mouse Ensembl browser. **(B)** Upper and lower band from the PCR product and sequenced. Sequence of upper and lower band aligned with *Rose.* Boxed area indicates the absence of NCE2 in *Rose* variant 2 (Lower band) **(C)** *Gapdh mRNA* was used as a loading control in Fig. 1B .

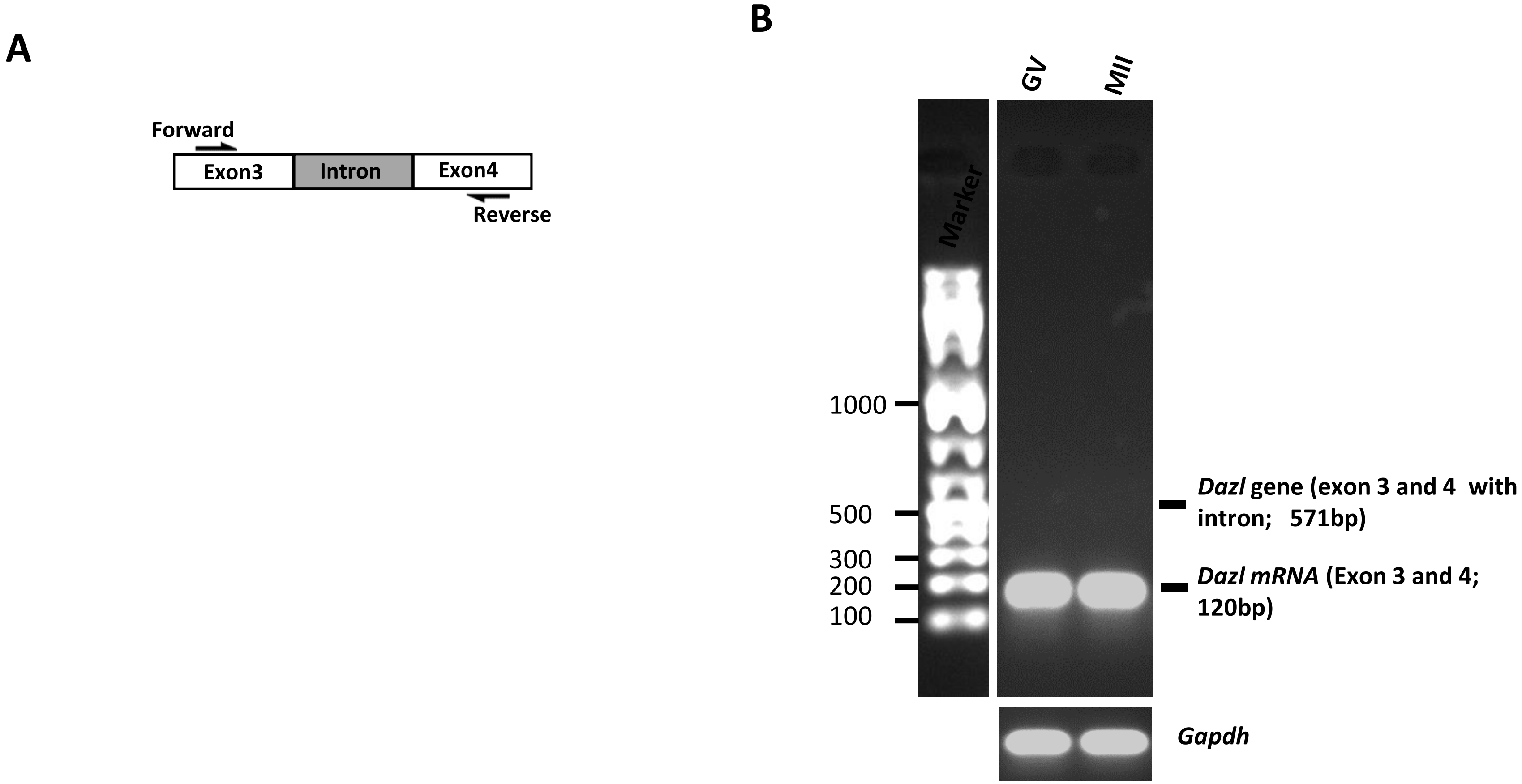

Supplementary Fig. 3: Isolated RNA do not contain DNA contamination. (**A**) Scheme of primer set up for detection of *Dazl* gene in the purified RNA sample of transcriptionally inactive GV and MII oocytes. (**B**) PCR analysis of processed *Dazl* mRNA showing absence of intron. *Gapdh mRNA* was used as a loading control.

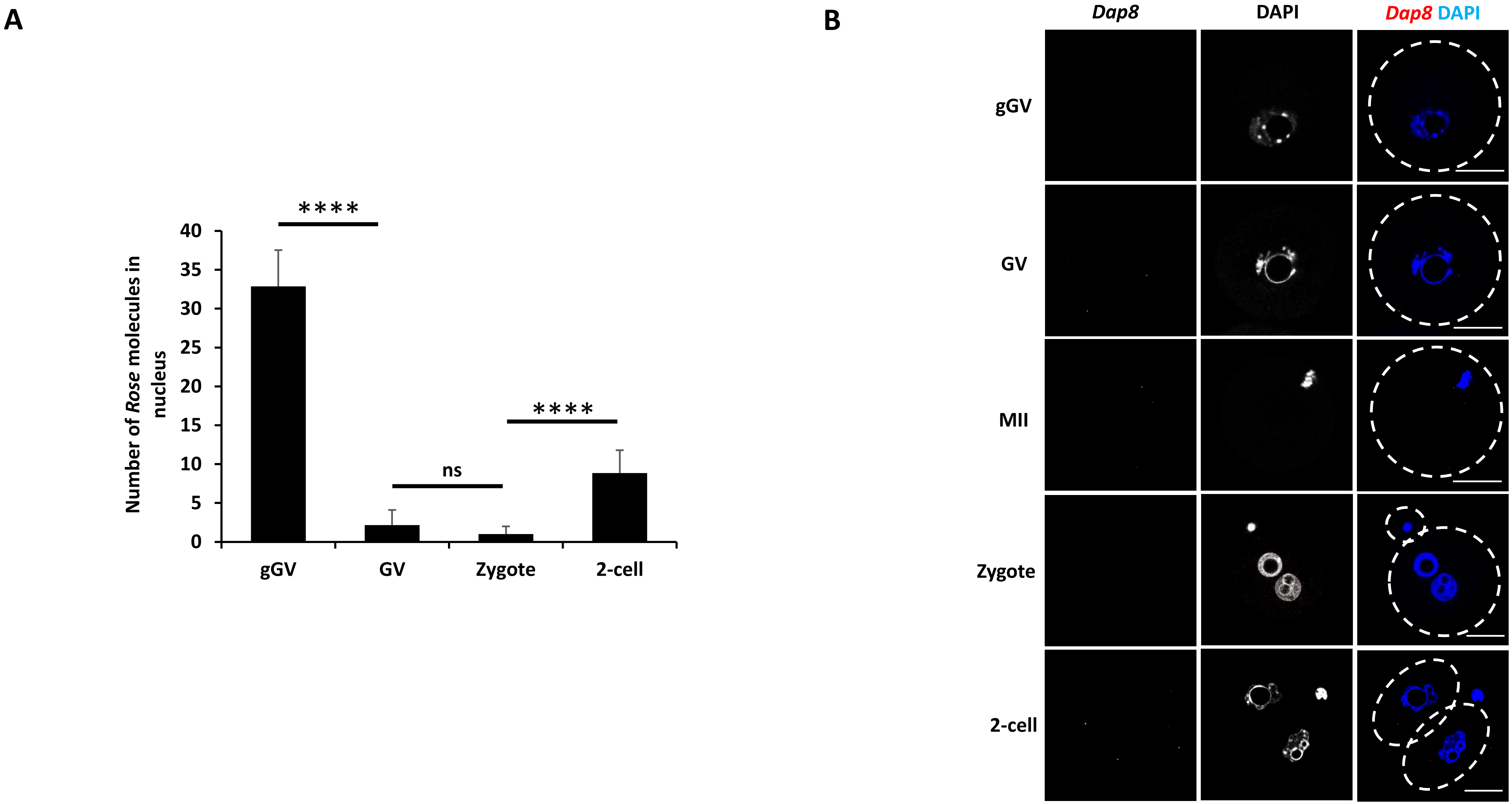

Supplementary Fig. 4: Expression of *Rose* lncRNA significantly decreases during developmental stages and is localized to nucleus of growing oocyte and 2-cell embryo. (**A**) Quantification of *Rose* lncRNA presence in the nucleus of growing and fully grown oocyte, zygote and 2-cell embryo. Mean ± SD; One-way ANOVA: F (3, 38) = 246,1, $p<0.0001$. Tukey's multiple comparisons test: ****$p<0.0001$, ns - non-significant; from three biological replicates, n≥7. (**B**) Bacterial *Dab8* RNA was used as a negative control for RNA FISH.

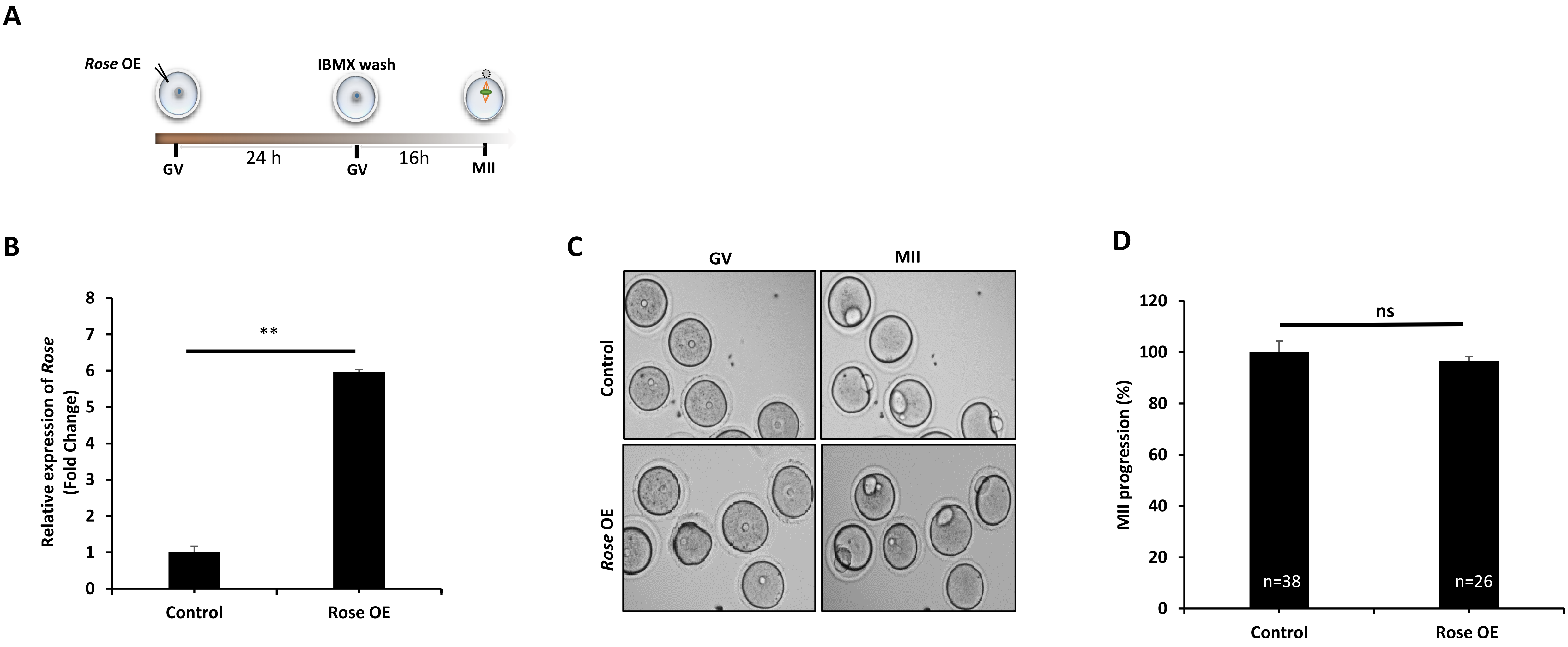

Supplementary Fig. 5: Overexpression of *Rose* does not affect meiotic progression. (**A**) Scheme of experimental approach for *Rose* overexpression. (**B**) qRT-PCR detection of overexpression of *Rose* lncRNA in GV oocytes. Mean ± SD; Student's t-test: **p<0.01 ; n=3. (**C**) Phenotype analysis of progression of GV oocytes to MII stage after overexpression of *Rose*; n≥18. (**D**) Quantification of oocyte progression from GV to MII stages after overexpression of *Rose*. Mean ± SD; Student's t-test: ns – non-significant; from three biological replicates, n≥18.

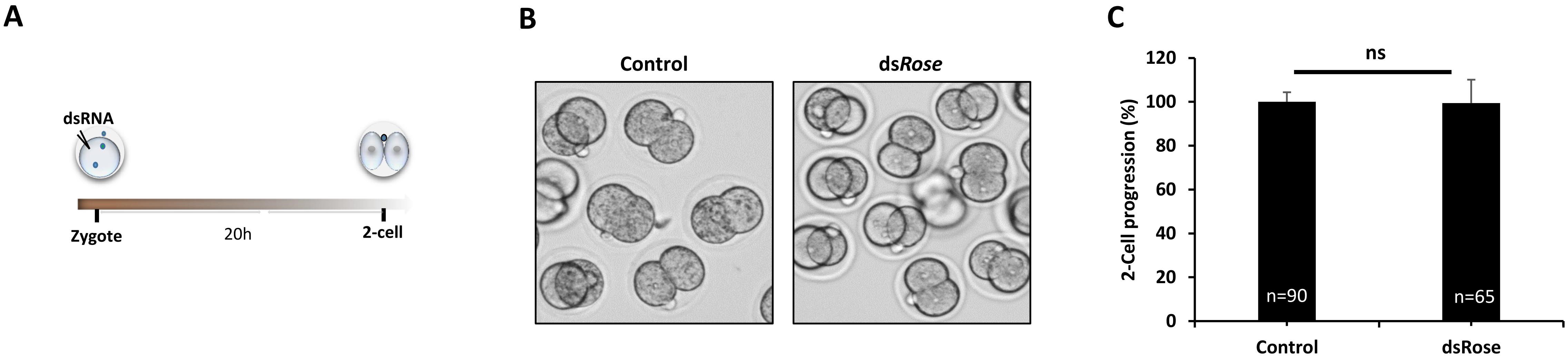

Supplementary Fig. 6: Downregulation of *Rose* does not affect 2-cell progression. (**A**) Scheme of experimental approach for *Rose* downregulation. (**B**) Phenotype analysis of progression of zygote to 2-cell embryo after downregulation of *Rose* lncRNA. (**C**) Quantification of 2-cell progression from zygote after downregulation of *Rose* lncRNA. Mean ± SD; Student's t-test: ns - non-significant; n≥12, from three biological replicates.

Primers

Name	Sequence 5'----- 3'	Amplicon Size (bp)
Upstream of NCE1 -F	AGTTGGTCCTTATAGAGGGC	682
Upstream of NCE1-R	GCATGAGATGATTCTGGTGT	
Downstream of NCE3 -F	CAGTTACCAGTGAACCAAGC	723
Downstream of NCE3 -R	CTTCAGAGCAAGGACACATA	
Rose NCE1-F	TCCAGGTTCTTCTTACAATG	482/594
Rose NCE3-R	CCAAAAACAGACTTGATGAG	
*T7*NCE1-F	CGAGTAATACGACTCACTATAGGTCCAGGTTCTTCTTACAATG	482/594
*T7*NCE3-R	CGAGTAATACGACTCACTATAGGCCAAAAACAGACTTGATGAG	
Rose-F	CTACGGCAGGAGTGACCCTA	256 (NCE1 region)
Rose-R	GGAGGGGGCTGTCTATGAAAAA	
Dazl exon 3-F	GTTTCTTTGCCAGATATGGCT	120
Dazl exon 4-R	CTACTATCTTCTGCACATCCAC	
18S-F	5'-CGCTCCACCAACTAAGAACG-3'	110
18S-R	5'-CTCAACACGGGAAACCTCAC-3'	
28S-F	5'-CTAAATACCGGCACGAGACC-3'	88
28S-R	5'-TTCACGCCCTCTTGAACTCT-3'	
GAPDH-F	TGGAGAAACCTGCCAAGTATG	130
GAPDH-R	GGTCCTCAGTGTAGCCCAAG	

RNA FISH Probes

Gene name	Target region	No. of ZZ pairs	channel	Cat. No.
Gm32743 (XR_379793.2)	2092 - 3137	20	C1	320269
Dab8 (EF191515)	414 - 862	10	C1	310043

Supplementary Table 1 : Primers and RNA FISH probes used in the study.

Name	Acccession #	RNA size	ORF size	Ficket Score	Hexamer Score	Coding Probability	Coding Label
Xist	NR_001570.2	12250	519	0.4809	-0.601593733	0.043572362	**no**
Ccnb1	NM_172301.3	2316	1293	1.143	0.233312892	0.999193219	**yes**
Rose	XR_379793.3	1611	240	0.4575	-0.187956336	0.030229861	**no**

Supplementary Table 2: Analysis of coding potential of *Rose* RNA using *CPAT* (http://lilab.research.bcm.edu/cpat/). Known *Xist* lncRNA and *Ccnb1* mRNA candidates was used as a controls.

Serial No.	Sum of Energy	Min of Energy	Transcript ID	Transcript Name	Biotype
1	-456.31	-23.85	ENSMUST00000219061	AC160249.1-201	Noncoding
2	-330.44	-21.09	ENSMUST00000113862	Kcnj15-208	Protein Coding
3	-304.64	-18.83	ENSMUST00000208147	Olfr2-203	Protein Coding
4	-259.72	-19.21	ENSMUST00000129791	Gm11373-201	Noncoding
5	-181.77	-18.28	ENSMUST00000053760	Utp14b-201	Protein Coding
6	-165.94	-21.30	ENSMUST00000110003	Eif4e1b-201	Protein Coding
7	-164.64	-17.70	ENSMUST00000095767	Etv1-201	Protein Coding
8	-147.66	-21.41	ENSMUST00000053880	Grin2b-201	Protein Coding
9	-135.67	-17.76	ENSMUST00000166353	Gm17402-201	Protein Coding
10	-128.84	-19.87	ENSMUST00000070342	Sertm1-201	Protein Coding

Supplementary Table 3: The top 10 interaction with *Rose* analyzed by LncRRIsearch web tool (http://rtools.cbrc.jp/LncRRIsearch/index.cgi).
